# Barriers and determinants of over-the-counter antibiotic sales de-escalation: perspectives of community pharmacists in Aseer, Saudi Arabia

**DOI:** 10.3389/fmed.2025.1677246

**Published:** 2025-09-22

**Authors:** Sultan M. Alshahrani, Sirajudeen Shaik Alavudeen

**Affiliations:** Department of Clinical Pharmacy, College of Pharmacy, King Khalid University, Abha, Saudi Arabia

**Keywords:** antimicrobial stewardship, antimicrobial resistance (AMR), over-the-counter antibiotics, community pharmacists, pharmacy practice, de-escalation, barriers

## Abstract

**Background:**

Antimicrobial resistance (AMR) represents a significant global health threat, which is worsened by the inappropriate distribution of antibiotics without a prescription. Cultural conventions and the easy accessibility of pharmacies in Saudi Arabia further aggravate this situation. Community pharmacists play a crucial role in antimicrobial stewardship, yet they continue to face substantial challenges.

**Objective:**

The objective of this study was to examine the barriers and determinants that influence community pharmacists’ capability to reduce over-the-counter (OTC) antibiotic sales in the Aseer region of Saudi Arabia.

**Methods:**

A cross-sectional survey was conducted with 269 community pharmacists, utilizing a validated, self-administered questionnaire. Data were analyzed using descriptive statistics, Chi-square tests, Kruskal-Wallis tests, and ordinal logistic regression to assess associations between demographic characteristics and pharmacists’ views of obstacles and stewardship practices.

**Results:**

The type of pharmacy was significantly associated with perceived barriers (*p* = 0.027), with chain pharmacists reporting more challenges than independent pharmacists. Logistic regression also showed that working in a chain pharmacy (OR = 2.01) and holding a PharmD degree (OR = 1.56) were predictors of higher perceived barriers. These findings suggest that both organizational factors and educational background shape how pharmacists perceive obstacles to reducing inappropriate antibiotic dispensing. Other demographic variables lacked statistical significance.

**Conclusion:**

The study highlights that systemic and educational factors strongly influence pharmacists’ roles in antimicrobial stewardship. This indicates that targeted interventions such as continuous professional education, digital prescription monitoring, and stronger regulatory enforcement are necessary to support pharmacists and reduce the dispensing of inappropriate antibiotics. These measures also align with Saudi Arabia’s Vision 2030 healthcare priorities. To our knowledge, this is the first study to investigate these barriers within the Aseer region, offering novel evidence on how organizational and educational contexts shape stewardship practices. The findings provide insights that extend beyond Saudi Arabia, informing global antimicrobial resistance containment strategies.

## Introduction

Antimicrobial resistance (AMR) represents a substantial worldwide health risk, compromising the effectiveness of antibiotics and elevating morbidity, mortality, and healthcare expenditures. The World Health Organization (WHO) projects that AMR may result in 10 million fatalities each year by 2050 if not addressed (25). The misuse of antibiotics, especially via over-the-counter (OTC) sales and self-medication, is a significant contributor to AMR, observed in both developed and developing countries (1). Cultural behaviors, easy access to pharmacies, and patient expectations in Saudi Arabia intensify this problem, with research indicating that 60–80% of patients request medications for viral infections (2). Community pharmacists, as primary healthcare practitioners, are crucial in managing antibiotic distribution and educating the public, but encounter substantial obstacles in reducing OTC antibiotic sales. This study examines these obstacles and determinants in the Aseer region, emphasizing the role of pharmacists in advancing Saudi Arabia’s Vision 2030–aligned healthcare reforms and national AMR containment strategies (3).

The improper distribution of antibiotics without prescriptions, commonly described as over-the-counter (OTC) antibiotic sales, is a prevalent problem in Saudi Arabia. Nafisah et al. (4) reported that 78% of pharmacists in Makkah dispensed antibiotics without prescriptions, driven by patient demand and financial incentives. Saha et al. (5) found that 65% of pharmacists experienced patient pressure to provide antibiotics, especially in chain pharmacies characterized by high customer volumes and sales targets. Another study noted that urban chain pharmacies are frequent sites for dispensing medications without prescriptions, with 45% of pharmacists attributing this practice to financial incentives (6). These behaviors contribute to the overuse of antibiotics, particularly broad-spectrum drugs, and promote the emergence of multidrug-resistant organisms (7). In 2018, the Saudi Ministry of Health (MoH) introduced a prescription-only law to curb OTC antibiotic sales, resulting in a 23.2% decrease in total antimicrobial sales and a 70% reduction in amoxicillin sales from 2017 to 2019 (7). However, sales of “Watch” medicines, including amoxicillin/clavulanic acid, increased by 6.5%, reflecting incomplete adherence and a shift toward broader-spectrum antibiotics, potentially worsening AMR (7). AlRukban et al. (8) highlighted that antibiotics are frequently dispensed in response to inappropriate requests, as only 36.7% of pharmacists consistently required valid prescriptions.

Systemic and cultural barriers hinder effective antibiotic stewardship. The lack of integrated patient tracking systems and incomplete prescription information, reported by 60% of pharmacists (5), complicates adherence to regulations. Other studies have also shown that inadequate infrastructure and weak physician–pharmacist communication undermine stewardship efforts globally (9). Culturally, self-medication is widespread in Saudi Arabia, with Al-Rasheed et al. (2) reporting that 65% of patients self-medicate due to easy accessibility and trust in pharmacists. Essack et al. (1) emphasized that community pharmacists are well-positioned to lead stewardship through patient education and enforcement of prescription policies, but they require training and systemic support. AlRukban et al. (8) found that only 19.5% of pharmacists actively counsel patients to prevent misuse; however, lack of time and limited privacy often restrict these initiatives.

The perspectives and preparedness of pharmacists regarding stewardship are essential. Saleh et al. (9) discovered that advanced training enhances adherence to antibiotic dispensing standards, especially among PharmD graduates. Haddadin et al. (6) observed a 25% decrease in non-prescribed dispensing following the 2018 regulation; however, adherence remains inconsistent due to enforcement deficiencies. Saha et al. (5) emphasized the need for compulsory AMR training to strengthen pharmacists’ contributions to public education and compliance with legislation. The 2018 rule corresponds with Vision 2030’s objective to enhance healthcare systems; nonetheless, its efficacy relies on overcoming obstacles such as patient awareness and pharmacy infrastructure (7).

Despite these regulatory advancements, existing studies in Saudi Arabia have primarily focused on antibiotic sales trends or general pharmacist practices, with limited attention to regional variations such as those in the Aseer region. Few studies have examined how systemic and cultural obstacles to antimicrobial stewardship interact with regional health reforms such as Saudi Arabia’s Vision 2030, despite the fact that international research has highlighted these obstacles. This gap restricts understanding of how community pharmacists perceive and respond to barriers under evolving regulatory and healthcare contexts. The novelty of the present study lies in its regional focus and in examining organizational and educational factors such as pharmacy type and pharmacist training that shape stewardship practices. Therefore, this study aims to investigate the specific barriers faced by community pharmacists in the Aseer region, with the hypothesis that demographic, organizational, and educational characteristics significantly influence their perceptions of obstacles to reducing inappropriate antibiotic dispensing, thereby providing evidence to support national and global AMR stewardship efforts.

## Materials and methods

### Study design

This research utilized a cross-sectional design, employing a questionnaire as the principal instrument for data collection. The study was conducted among community pharmacists in the Aseer region of Saudi Arabia. Participants independently completed the questionnaire, ensuring anonymity and confidentiality of responses. Participation was voluntary, and informed consent was obtained before survey initiation.

### Study population

The questionnaire was distributed to 300 community pharmacists in the Aseer region using a convenience sampling approach, rather than a true randomization method, as participation depended on the pharmacists’ availability and willingness to respond. Each selected pharmacy typically employed 2–3 pharmacists working in two shifts. According to recent statistics, approximately 747 registered community pharmacists in Aseer were eligible to participate (10). The minimum sample size of 252 was estimated using a standard sample size formula based on a 95% confidence interval, a 5% margin of error, and a 50% response distribution (11). The study ultimately included 269 community pharmacists, ensuring adequate representation. The overall response rate was 89.7%, as some pharmacists declined or did not complete the survey, which may have introduced non-response bias. To minimize this risk, follow-up reminders were sent electronically during the data collection period. The inclusion criteria were licensed community pharmacists working in the Aseer region, while interns, unlicensed staff, and incomplete responses were excluded from the analysis.

### Study tool and data collection

The questionnaire was developed to align with the study’s objective of exploring barriers and determinants of de-escalating over-the-counter (OTC) antibiotic sales, drawing on previous studies (1, 2, 4–9). It comprised four domains: Domain I (6 items) captured demographic characteristics, including age, gender, educational background, years of experience, pharmacy type (chain vs. independent), and location (urban vs. rural); Domain II (8 items) assessed perceptions of barriers to reducing OTC antibiotic sales, such as patient self-medication and systemic gaps; Domain III (6 items) evaluated determinants like continuous education and antimicrobial stewardship; Domain IV (6 items) explored pharmacists’ roles in AMR containment and alignment with Vision 2030. Across these domains, items were structured as a mix of Likert-scale questions (five-point), dichotomous (yes/no), and multiple-choice formats.

A pilot study was carried out involving 20 community pharmacists in Abha, Aseer, to ensure both reliability and validity. The pilot evaluated clarity, relevance, and internal consistency, yielding a Cronbach’s alpha of 0.81, indicating robust reliability. Content and face validity were confirmed by three senior pharmacy practice professionals, who reviewed the items for appropriateness and coverage. Their structured feedback was incorporated into the final version of the questionnaire. The pilot data were excluded from the final analysis.

The questionnaire was created using Google Forms and electronically disseminated to pharmacists in Abha, Khamis Mushait, and other prominent cities in Aseer from July to October 2024, which constituted the full duration of data collection for this study. Participation was optional, and pharmacists were not obligated to provide their identity or the location of their pharmacy. Inquiries regarding the survey were promptly addressed and resolved. Data confidentiality was protected via restricted access to a limited study team. Because the survey was distributed exclusively online, pharmacists without reliable internet access or adequate digital literacy may have been underrepresented. However, online dissemination was chosen for its feasibility, wide reach, and ability to maintain confidentiality. The full questionnaire is provided as a Supplementary Appendix for reference.

### Statistical analysis

The completed surveys were examined for precision and completeness. Incomplete or missing responses were excluded listwise from the analysis to maintain data integrity, and sensitivity checks confirmed that excluded cases did not significantly alter overall results (12). The data were encoded and input into SPSS version 24 (IBM Corp., Armonk, NY, United States).

Descriptive statistics, encompassing frequencies and percentages, encapsulated demographic factors such as age, gender, education, pharmacy type, geography, and experience. Responses to Likert-scale items regarding barriers, determinants, and pharmacists’ roles were classified as ordinal data, with medians and interquartile ranges (IQRs) utilized to convey core tendencies and variability. Normality of continuous variables was tested using the Shapiro–Wilk test (13), and the results supported the use of non-parametric statistics for skewed data.

The Chi-Square Test (χ^2^) of Independence analyzed the relationships between demographic characteristics (e.g., gender, pharmacy type, location) and outcomes, such as high versus low perceived barriers or support for stewardship, with a *p*-value < 0.05 indicating statistical significance. The Kruskal-Wallis H Test was employed to compare views among demographic categories (e.g., age, education, experience), appropriate for non-normally distributed ordinal data. When significant differences were observed, *post hoc* pairwise comparisons were performed using Dunn’s test with Bonferroni correction to control for multiple testing (14).

Ordinal logistic regression determined predictors of elevated perceived barriers and diminished stewardship support, utilizing gender, age, education, pharmacy type, location, and experience as independent variables. All assumptions for ordinal regression, including proportional odds and absence of multicollinearity, were verified before analysis (15). The threshold for statistical significance was established at *α* < 0.05.

### Ethical approval and informed consent statements

The study received approval from the Research Ethics Committee of King Khalid University (ECM# 2021-3606). Informed consent was obtained from all participants before data collection.

## Results

### Demographic characteristics

This research included 269 community pharmacists from the Aseer region. The predominant demographic of participants was male (64%). The majority were recently graduated, with 56% falling within the age range of 20–29 years. Pharmacists aged 30–39 represented 29.7%, and those aged 40 and older were 14.3% of the sample. Most respondents held a PharmD degree (71%), followed by bachelor’s degrees (22.3%) and postgraduate certificates (6.7%). A significant majority (89%) were employed in chain pharmacies, whereas 11% were engaged in independent community pharmacies. Seventy-nine percent of participants practiced in urban regions. Regarding professional experience, 46% had less than 5 years, 33.5% had between 6 and 10 years, and 20.5% had more than 11 years. In addition to categorical frequencies, the median age of respondents was 28 years (IQR = 25–34), and the median years of experience was 5 years (IQR = 2–9). Table 1 summarizes the demographic characteristics of participants.

**Table 1:** Demographic characteristics of participants (*N* = 269).

Characteristic	Category	Frequency (*n*)	Percentage (%)
Gender	Male	172	64.0%
	Female	97	36.0%
Age group (years)	20–29 years	151	56.0%
	30–39 years	80	29.7%
	40+ years	38	14.3%
Education level	Bachelor’s degree	60	22.3%
	PharmD	191	71.0%
	Postgraduate degree	18	6.7%
Pharmacy type	Chain pharmacy	239	89.0%
	Independent pharmacy	30	11.0%
Location	Urban	212	79.0%
	Rural	57	21.0%
Years of experience	0–5 years	124	46.0%
	6–10 years	90	33.5%
	11+ years	55	20.5%

Summary statistics (Median, IQR): Age = 28 (25–34); Years of experience = 5 (2–9).

### Perceived barriers to antibiotic stewardship

**Figure 1:**
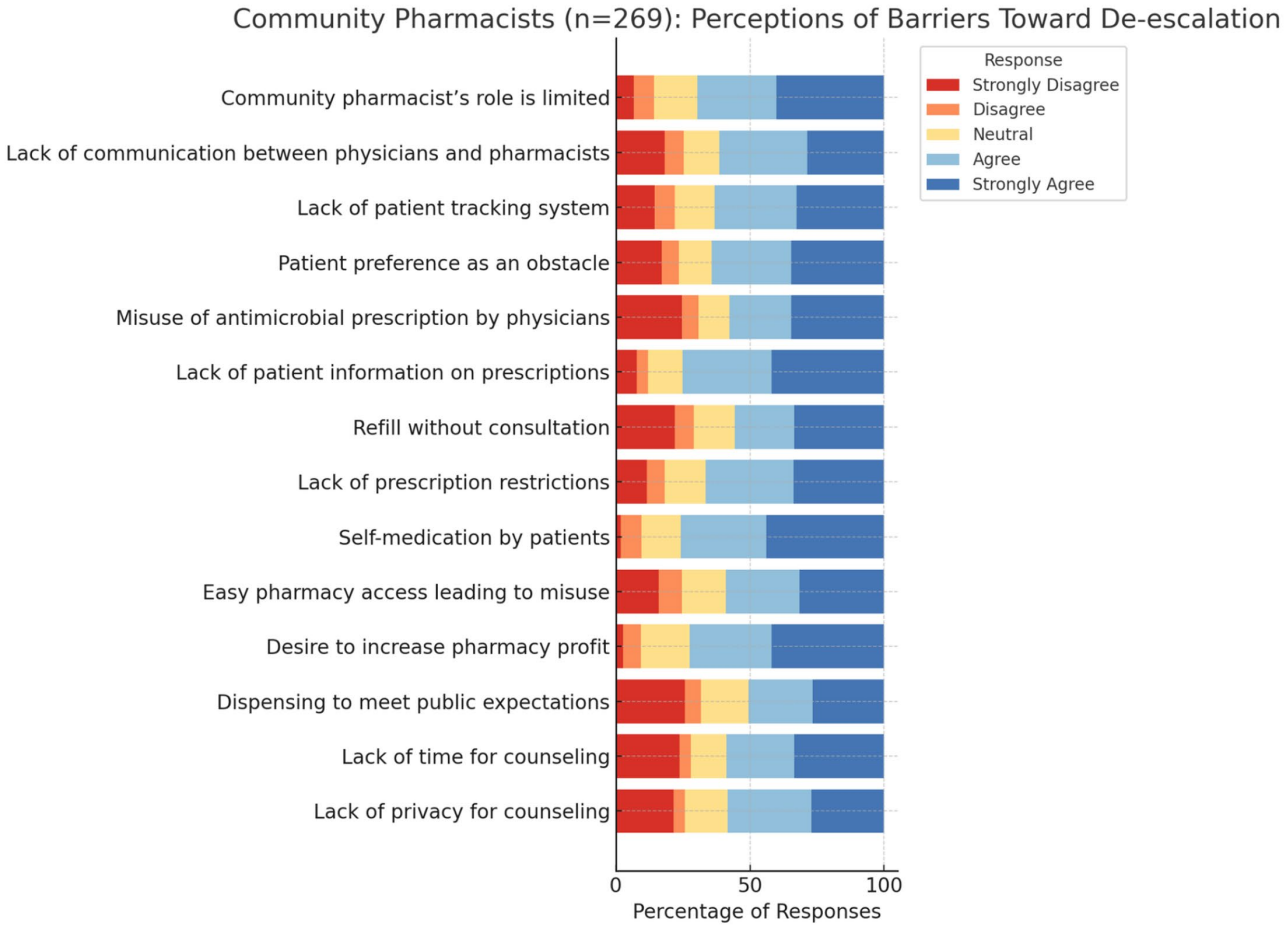
Perceived barriers to antibiotic stewardship.

Pharmacists identified multiple barriers that hinder efforts to restrict OTC antibiotic sales. Patient demand was a major issue, with 64.3% reporting that patients often preferred antibiotics without prescriptions. Similarly, 66.5% cited the lack of rigorous prescription regulations as a challenge, and 50.6% noted social expectations for rapid access to antibiotics. Financial incentives also played a role, with 72.5% acknowledging that profit motives conflicted with stewardship practices. These findings highlight how patient expectations, weak enforcement, and financial pressures interact to perpetuate inappropriate antibiotic dispensing. Supplementary Table and Figure 1 illustrate that patient self-medication and ease of access to pharmacies were perceived as the most influential drivers of misuse.

### Pharmacists’ perspectives on stewardship, regulations, and public awareness

Community pharmacists expressed strong support for stewardship and regulations aimed at curbing inappropriate antibiotic use. The highest consensus was observed for pharmacist involvement in stewardship programs (median = 5, IQR = 4–5). Support for Ministry of Health regulations also ranked highly (median = 4, IQR = 2–5). Continuous professional education was emphasized (median = 4, IQR = 3.0). Public awareness efforts were rated as insufficient (median = 4, IQR = 3–5), while insured patients were frequently perceived as receiving unnecessary antibiotics (median = 4, IQR = 3–5). Perspectives on primary healthcare centers were mixed (median = 3, IQR = 2–4). All IQR values were reported in Table 2.

**Table 2:** Pharmacists’ perspectives on stewardship, regulations, and public awareness (Median and IQR: Q1-Q3).

Item	Median	IQR (Q1–Q3)
Community pharmacists need continuous education	4	3–5
MoH regulations beneficial	4	3–5
Physicians easily prescribe to insured patients	4	2–5
Awareness programs for the public are insufficient	4	2–5
Role of PHC centers needs improvement	3	2–4
Pharmacists should engage more in stewardship programs	5	4–5

IQR, interquartile range (25th–75th percentile). Responses measured on a 5-point Likert scale (1 = strongly disagree, 5 = strongly agree).

### Inferential analysis of demographic factors and stewardship perceptions

The Chi-square test examined associations between demographic factors and perceived barriers (Table 3). “High barriers” were defined as scores ≥ the median, while “low barriers” were scores < the median. Pharmacy type showed a statistically significant correlation with perceived barriers (χ^2^ = 4.89, *p* = 0.027), with chain pharmacists reporting greater difficulties. No significant associations were found for gender (*p* = 0.143) or location (*p* = 0.081).

**Table 3:** Association between demographic characteristics and perceived barriers.

Demographic variable	High barriers (*n*, %)	Low barriers (*n*, %)	χ^2^	*p*-value
Gender	96 (55.8%)	76 (44.2%)	2.14	0.143
Pharmacy type	152 (63.6%)	87 (36.4%)	4.89	0.027^*^
Location	162 (76.4%)	50 (23.6%)	3.05	0.081

^*^p < 0.05, statistically significant.

The Kruskal–Wallis H test compared perceptions across age, education, and experience (Table 4). Significant differences were observed among age groups (H = 6.48, *p* = 0.039) and educational levels (H = 4.12, *p* = 0.043), with younger pharmacists and PharmD graduates reporting greater awareness of barriers. Variations based on years of experience were not statistically significant (*p* = 0.088). Dunn’s *post hoc* tests with Bonferroni correction identified the specific group differences.

**Table 4:** Comparison of perceived barriers across age, education, and experience.

Variable	Mean rank (high barriers)	Mean rank (low barriers)	H statistic	*p*-value
Age group	145.2	127.6	6.48	0.039^*^
Education level	141.8	123.3	4.12	0.043^*^
Years of experience	138.4	129.5	2.91	0.088

^*^p < 0.05, statistically significant.

### Regression analysis

Ordinal logistic regression identified key predictors of perceived barriers (Table 5). Pharmacists in chain pharmacies were more likely to report higher barriers (OR = 2.01, *p* = 0.019), and PharmD degree holders also reported greater restrictions (OR = 1.56, *p* = 0.041). Other demographic variables were not significant. Model diagnostics indicated acceptable fit (Hosmer–Lemeshow *p* > 0.05), adequate explained variance (Nagelkerke R^2^ = 0.21), and no evidence of multicollinearity.

**Table 5:** Ordinal logistic regression predicting high perceived barriers and low stewardship support.

Predictor variable	Odds ratio (OR)	95% Confidence interval (CI)	*p*-value
Male gender (vs. female)	1.24	0.82–1.89	0.308
Chain pharmacy (vs. independent)	2.01	1.12–3.58	0.019^*^
Urban location (vs. rural)	1.43	0.91–2.25	0.120
Age 30–39 (vs. 20–29)	0.81	0.50–1.30	0.379
Age 40 + (vs. 20–29)	0.69	0.39–1.24	0.210
PharmD (vs. bachelor’s)	1.56	1.02–2.39	0.041^*^
Postgraduate (vs. bachelor’s)	1.24	0.58–2.65	0.577
Experience 6–10 years (vs. < 5 years)	1.12	0.69–1.82	0.641
Experience 11+ years (vs. < 5 years)	0.92	0.51–1.68	0.783

^*^p < 0.05, statistically significant.

## Discussion

Antimicrobial resistance (AMR) continues to pose a global health problem, severely compromising the efficacy of current therapeutic measures. Misuse of antimicrobials, particularly the over-the-counter distribution of antibiotics, has been a principal catalyst of this problem (7, 16). Saudi Arabia has reported elevated levels of antimicrobial usage, prompting the need for regulatory measures to mitigate misuse and enhance stewardship. The implementation of the national antimicrobial prescription-only policy in 2018 marked a significant advancement in Saudi Arabia’s initiatives to address AMR. Subsequent to this policy, multiple studies demonstrated significant decreases in antibiotic sales. Alajmi et al. (7) reported a 23.2% decrease in retail antimicrobial sales from 2017 to 2019, highlighting the prompt effects of regulatory enforcement. Alzahrani et al. (16) observed a 9–10% reduction in antimicrobial usage rates in community pharmacies (7, 16).

Community pharmacists, as essential stakeholders identified by our study findings, predominantly viewed the law favorably. Alakhali et al. (17) reported that almost 90% of pharmacists agreed that implementing the prescription-only policy would help decrease inappropriate antimicrobial usage. A study by Alrasheedy et al. (18) corroborated this finding, demonstrating that the percentage of pharmacies distributing antibiotics without prescriptions dramatically decreased after the implementation of law enforcement measures (17, 18). Our own regression results support this, showing that pharmacists in chain pharmacies were twice as likely to report higher barriers (OR = 2.01, 95% CI: 1.12–3.62), while PharmD graduates were 1.56 times more likely (95% CI: 1.02–2.39) to recognize stewardship challenges compared to bachelor’s degree holders.

Despite these positive advancements, numerous difficulties persist. Full adherence to the policy has not yet been attained. Alhomoud et al. (19) discovered that antibiotics are still dispensed without prescriptions in certain community pharmacies, frequently influenced by patient requests, economic incentives, or misunderstandings about the need for medical consultations for infections. Another study identified systemic hurdles such as financial incentives, patient expectations, and inadequate regulatory enforcement as significant obstacles to complete compliance (7). Our findings confirm these challenges, with financial motives and patient pressure ranked among the highest barriers reported by pharmacists.

The policy has inadvertently altered antibiotic prescribing practices. Sales of narrow-spectrum antibiotics, such as amoxicillin, have declined, whereas broader-spectrum medicines, like amoxicillin/clavulanic acid, have had an uptick in sales (7). This tendency, albeit indicative of enhanced regulatory compliance, introduces new concerns for AMR by potentially fostering the selection of resistant species.

The intricacy of AMR mitigation is exacerbated by cultural and infrastructural elements. Raju et al. (20) indicated that hardly one-third of pharmacists consistently solicit genuine prescriptions, highlighting persistent deficiencies in practice. Cultural attitudes that endorse self-medication and reliance on pharmacists as primary providers persist in Saudi society (8, 14). Moreover, infrastructural constraints, such as the lack of integrated computerized prescribing and patient tracking systems, impede the complete achievement of stewardship objectives (5).

In response to these issues, Saudi Arabia has established an extensive National Action Plan for AMR (2022–2025), in accordance with the World Health Organization’s Global Action Plan. This approach focuses on fortifying regulatory frameworks, advancing public and professional education, improving monitoring systems, and optimizing antibiotic utilization in both human and animal health sectors (21).

Our study contributes to this context by demonstrating that organizational (pharmacy type) and educational (PharmD vs. bachelor’s) characteristics significantly influence pharmacists’ perceptions of barriers. Specifically, chain pharmacists reported higher barriers due to organizational pressures such as profit-driven business models and sales targets, while PharmD graduates—trained more extensively in stewardship—demonstrated heightened awareness of inappropriate dispensing practices. These interpretations are consistent with evidence from Saudi Arabia and the Gulf, where commercial consolidation has been linked to increased patient load and sales-driven dispensing pressures (18, 19). These findings emphasize that, beyond regulation, workplace dynamics and professional training strongly influence stewardship outcomes.

Pharmacists are particularly equipped to play a pivotal role in the efficacy of antimicrobial resistance containment efforts. Their accessibility and function as primary health advisors allow them to implement prescription rules, inform patients about the dangers of improper antibiotic usage, and advocate for safe practices (20). For pharmacists to excel in this role, continuous professional development, supportive regulatory frameworks, and incentive models that discourage profit-driven dispensing in chain settings are essential. This aligns with global evidence from systematic reviews, which demonstrate that clinical pharmacists play a central role in ASPs through prospective audit, feedback, and educational interventions, directly improving antimicrobial use and stewardship outcomes (22).

Comparisons with international studies suggest that these challenges are not unique to Saudi Arabia. For example, evidence from Egypt indicates that inappropriate antibiotic dispensing is prevalent in community pharmacies, often due to patient demand and inadequate regulation (23). In Jordan, Haddadin et al. (6) documented that nearly 40% of community pharmacies dispensed antibiotics without prescriptions, highlighting organizational and regulatory gaps. Qualitative work by Saleh et al. (23) further emphasized structural barriers, such as commercial pressures and a lack of stewardship training, as key challenges for pharmacists. A recent systematic review of pharmacist-led antimicrobial stewardship interventions in low-and middle-income countries further supports these findings, showing that organizational pressures and limited resources commonly hinder optimal stewardship, yet targeted pharmacist-driven interventions significantly improve prescribing practices (24). These parallels situate the Aseer findings within the broader LMIC and GCC context, strengthening their relevance to global AMR stewardship efforts.

Future initiatives must emphasize ongoing pharmacist education regarding antimicrobial stewardship principles, stricter enforcement of prescription regulations, the establishment of national digital prescribing systems, and public awareness campaigns designed to alter societal perceptions of antibiotics. Furthermore, comprehensive monitoring and evaluation frameworks are crucial to guarantee that regulatory interventions result in enduring behavioral modifications among both providers and patients. Community and chain pharmacies’ incentive models need to be adjusted to remove the connection between financial gain and antibiotic sales volume to specifically address structural barriers. Compulsory AMR-focused training and continuing education programs should be adapted to various qualification levels (PharmD vs. bachelor’s) in order to address educational barriers and guarantee that all pharmacists gain the skills required for stewardship. These recommendations align directly with Saudi Arabia’s Vision 2030 reforms, which place a high priority on improving primary care, digital health infrastructure, and the use of rational medicine.

This study possesses multiple shortcomings that warrant acknowledgment. A significant portion of the data concerning antimicrobial sales and pharmacist conduct was obtained via self-reported surveys, which may be influenced by social desirability bias and underreporting. Secondly, whereas national databases like IQVIA and SFDA sales statistics offer significant insights, they may not comprehensively account for informal or uncontrolled sales transpiring outside registered pharmacies. The research primarily concentrated on urban environments, which may restrict applicability to rural regions where the implementation of antimicrobial rules could vary. Moreover, the influence of external factors, including public health initiatives or seasonal fluctuations in infection rates, was not uniformly considered in the available data. Although alterations in prescribing patterns were seen, establishing causal links between policy enforcement and antimicrobial resistance trends necessitates additional longitudinal and microbiological surveillance to provide more robust evidence. These constraints underscore the necessity for more thorough and methodical research in subsequent assessments. Ultimately, the higher proportion of chain pharmacy participants reflects the actual distribution in urban areas of Aseer, where chains dominate due to commercial consolidation. Stratified sampling was not applied due to resource constraints, though future studies should ensure proportional representation of independent pharmacies to enhance generalizability. Therefore, sampling bias due to overrepresentation of chain pharmacies may limit generalizability, especially for rural or independent pharmacy perspectives.

## Conclusion

Antimicrobial resistance (AMR) represents an evolving risk to public health worldwide and in Saudi Arabia. This study is the first to explore barriers to the de-escalation of over-the-counter antibiotic sales in the Aseer region, providing novel regional insights into organizational and educational determinants of stewardship. Although many advancements have occurred since the implementation of the 2018 prescription-only policy, significant obstacles persist. Community pharmacists are essential in implementing antimicrobial stewardship; nevertheless, they face systemic obstacles such as patient expectations, commercial pressures, and insufficient regulatory support. Our findings demonstrate that PharmD graduates, due to their stronger stewardship training, reported greater awareness of barriers, while chain pharmacists perceived more challenges, likely reflecting higher patient volumes and profit-driven sales models.

The results underscore that, in addition to legal enforcement, a comprehensive strategy is essential. Practical next steps include mandatory AMR-focused continuing education for pharmacists, stricter enforcement of prescription-only dispensing laws, integration of national digital prescription monitoring systems, and restructuring of financial incentives in pharmacies to prioritize stewardship over sales. Enhanced coordination between pharmacists and physicians and targeted public awareness campaigns are also necessary to reduce self-medication behaviors.

These policy-level recommendations directly align with Saudi Arabia’s National Action Plan for AMR (2022–2025) and Vision 2030 healthcare reforms, particularly in strengthening primary care, expanding digital health infrastructure, and promoting rational medicine use. Future initiatives must also prioritize the enhancement of rural healthcare infrastructure to ensure equitable implementation across diverse settings. Policymakers can convert regulatory frameworks into long-lasting behavioral change by addressing both structural and educational barriers. Policymakers, healthcare professionals, and the general public can work together to make sustainable, measurable progress in reducing the misuse of antibiotics and preserving their efficacy for future generations.

## Data Availability

The original contributions presented in the study are included in the article/supplementary material, further inquiries can be directed to the corresponding author.
